# A Kantian ethics approach to moral bioenhancement

**DOI:** 10.1111/bioe.12380

**Published:** 2017-09-05

**Authors:** Sarah Carter

**Keywords:** empathy, enhancement, Kantian ethics, moral bioenhancement, moral enhancement

## Abstract

It seems, at first glance, that a Kantian ethics approach to moral enhancement would tend towards the position that there could be no place for emotional modulation in any understanding of the endeavour, owing to the typically understood view that Kantian ethics does not allow any role for emotion in morality as a whole. It seems then that any account of moral bioenhancement which places emotion at its centre would therefore be rejected. This article argues, however, that this assumption is incorrect. Given later writings by Kant on the role of sympathy, and taking into account other concerns in Kantian ethics (such as bodily integrity), it may in fact be the case that Kantian ethics would allow for an account of moral bioenhancement through emotional modulation, and that in some (rare) cases such an intervention might even be considered to be a duty.

## 1 INTRODUCTION

While moral bioenhancement is a contemporary field, Kantian ethics has a contribution to make to this emerging debate because of the Kantian duty to strive for moral perfection. A more in‐depth explanation of the term can be found below but, in short, moral perfection^1^ involves not only the ability to avoid being steered mindlessly by emotions and inclinations, but also cultivating those inclinations that aid us in behaving morally. The second part of this definition can certainly been seen having particular relevance for moral bioenhancement, especially when discussing the emotion‐centred approach to the endeavour, as not only does it seem to acknowledge a role for emotions in Kantian morality,^2^ it also presents a duty that moral bioenhancement could help agents to fulfil. For this reason I argue that Kantian ethics can enrich the debate about moral bioenhancement and in this article, I will explore and develop this idea.

There has been a long‐standing debate between cognitivists and non‐cognitivists as to whether morality is, at its core, a cognitive or emotional enterprise. One instance where this conflict is clearly represented is in the works of Kant (1724–1804) and Hume (1711–1776). Hume of course wrote that “reason alone can never produce any action, or give rise to volition”,^3^ and that it is emotion, not reason, which moves us to action. Meanwhile Kant, however, disagreed and instead spoke of the importance of rationality in morality: of its role both in evaluation of actions and duties and even in terms of motivation. As Denis and Wilson note: “Regarding moral motivation: It is a central feature of Kant's ethics that pure reason can be practical—that reason can “of itself, independently of anything empirical, determine the will.” (CPrR 5: 42).^4^

This debate continues to this day, and its discussion seems to have been further fuelled by the debate on moral bioenhancement^5^ in which writers such as Douglas^6^ and Persson and Savulescu^7^ argue in favour of emotional modulation as a method of enhancing a person's morality (the former arguing in favour of attenuating ‘non‐moral emotions’ and the latter arguing in favour of amplifying emotions such as empathy), whilst John Harris^8^ argues that the rational nature of morality means that to consider such a method to be *moral* enhancement would be nonsensical and so it would make more sense to speak of *cognitive* enhancement rather than moral enhancement.

Given the above, it might, at first glance, seem that a Kantian approach to moral enhancement would tend towards the position offered by Harris: that there could be no place for emotional modulation in any understanding of moral enhancement, as there could be no role for emotion in morality. I will argue, however, that this assumption is in fact incorrect: that given later writings by Kant on the role of sympathy, and also taking into account other concerns in Kantian ethics (such as bodily integrity), it may be the case that Kantian ethics could allow for an account of moral bioenhancement through emotional modulation,^9^ and that in some cases it might even be considered to be a duty.

In order to assert this position, I will explore various important Kantian concepts and arguments. As noted above, Kantian ethics is famously very focused on the rational basis of morality, and Kant is generally regarded as having taken the view that the emotions can be a hindrance to morality, rather than something to be encouraged or enhanced. In short, it might be thought that he believed them to have no such role. However, I will note that Kant's views on the role of emotions in morality shifted in later works, allowing a place in ethics for sympathy^10^ in particular; I will then consider this new possible role for emotion in morality within the context of moral bioenhancement. This being said, the use of biomedical interventions towards this end could be considered to be morally distinct from the more traditional methods of amplifying moral emotions that were proposed by Kant (namely exposing oneself to emotive stimuli), and so, taking into account Kant's assertion that we are necessarily embodied persons, at this juncture I will take some time to consider concerns that could be raised here regarding the possible impact of moral bioenhancement on the body, the mind, and even on identity, I will consider as well the Kantian argument of self‐preservation in the context of moral bioenhancement. Finally, I will then come to consider whether there could be said to be a duty^11^ to undergo moral bioenhancement, a question which will be explored with regards to the Kantian duty to strive for moral perfection. A more detailed explanation is found later in this article, but in brief, moral perfection for Kant involves cultivation of those inclinations that aid us in behaving morally, but further requires that we also avoid being steered mindlessly by emotions and inclinations.^12^ Kant regarded the duty to strive for moral perfection as imperfect in nature,^13^ this means that while the ends of the duty are set, its means are not and so we as agents are given free rein in how we act to accomplish those ends. This is in contrast to perfect duties where both the ends and the means of a duty are set and so agents are instructed in how to fulfil the duty in question.^14^

## 2 THE ROLE OF EMOTIONS

It is typically considered that a Kantian account of ethics would not allow for any moral emotion – enhanced or otherwise.^15^ However, as I have mentioned, the views of Kant himself on this matter shifted in later works (as I explain below), allowing a place for sympathy in his moral theory and leaving us open to question whether there could be room for moral bioenhancement in Kantian ethics.

It would be prudent to clarify that, while Kant speaks of sympathy in his writing, this is not to say that this would not also constitute a reference to empathy as well. Much of this is due to the era in which Kant lived, for the term ‘empathy’ only came into being at the start of the twentieth century, having been used as a translation for the German psychological concept of *Einfühlung* (or “feeling‐in”, which itself came into usage in the late nineteenth‐century).^16^ In addition to the matter of timing, it is important to note that definitions of empathy (while they can be rather varied)^17^ can often overlap with our modern understanding of sympathy, as Wispé notes: “To complicate matters, however, a number of other terms – sympathy, role taking, perspective taking, and so on – may or may not, refer to similar, or identical, psychological processes [as empathy].”^18^ Further, some writers suggest that empathy could even encapsulate sympathy as part of its own definition.^19^ It is beyond the scope of this article to delve too deeply into the relationship between empathy and sympathy, but it is important to note that while much of the language around moral bioenhancement (by emotional modulation) speaks of empathy, this is not at all at odds with the sympathy of which Kant speaks – indeed they may very well, both in principle and practice, be one and the same.

Returning to the matter at hand, perhaps the best known reading of Kant's views of emotions in morality is that which he espouses in his early writings, such as *Groundwork on the Metaphysics of Morals* where he makes his opinion of the worth of inclinations quite plain:

Inclinations themselves, as sources of needs, are so far from having an absolute value to make them desirable for their own sake that it must rather be the universal wish of every rational being to be wholly free from them. (G 428)^20^

Reading this, it seems that any question regarding the view that Kant might have taken of moral bioenhancement would be very quickly and easily answered. Moral enhancement by way of augmentation of those qualities on which Kant placed no value, and indeed spoke of all rational beings having a “wish… to be wholly free from them”, seems to be something with which Kant would take great issue.

But this is not necessarily the case, for, as De Lourdes Borges notes: “in the *Doctrine of Virtue*, Kant explicitly states that we should use sympathy as an incentive to benevolent actions when the sole respect for moral law is not sufficient.”^21^And due to its new‐found usefulness, Kant even goes so far as to say that we should cultivate sympathetic tendencies. As Baron notes:

In a passage from the *Doctrine of Virtue*…, Kant says that we are to cultivate our sympathetic feelings, and he suggests that we can do so by, for example, seeking out “places where the poor who lack the most basic necessities are to be found” (*MM* 457).^22^

The purpose of such an exercise would be to expose ourselves to emotions such as sympathy – which will likely be aroused at seeing pitiful and sad sights such as those that Kant describes – so that we might learn how to temper and control them at will and as necessary. Offering an interesting analogy, De Lourdes Borges explains:

Just as a medical student should be desensitized to blood before beginning to practice surgery, we should make our natural sympathetic emotions arise in order to be capable of controlling and using them on appropriate occasions.^23^

Indeed, it could be argued Kant had previously made some subtle reference to the idea of cultivating sympathy in morality through his views on man's duties to animals in his *Lectures on Ethics*. As Kant wrote:

So if a man has his dog shot, because he can no longer earn a living for him, he is by no means in breach of any duty to the dog, since the latter is incapable of judgement, but he thereby damages the kindly and humane qualities in himself, which he ought to exercise in virtue of his duties to mankind. Lest he extinguish such qualities, he must already practice a similar kindness towards animals; for a person who already displays such cruelty to animals is no less hardened towards men.^24^

However it is not entirely clear what role is played by sympathy. Some, like De Lourdes^25^ argue that its role is motivational, while Baron^26^ suggests that it is more to do with helping us to determine how best to fulfil our duties; Denis^27^ seems to consider it to be a combination of the two. In any case, regardless of whose interpretation as to the role of moral emotion in Kantian ethics is correct, there does seem to be a role for sympathy in Kantian ethics after all.

Even if we were to dismiss Kant's change of heart, to dismiss any direct role that sympathy could be said to have in a Kantian approach to morality, moral enhancement via emotional modulation could perhaps still prove useful in such an account. Kantian ethics does of course put reasoning at the heart of its moral theory, but there are times when certain emotions can become obstacles to moral reasoning and can cloud moral judgement. The emotions in question here are those which Douglas^28^ refers to as “counter‐moral emotions”, offering racial bias/aversion and aggression as examples of these. As he explains:

One example of a counter‐moral emotion might be a strong aversion to certain racial groups. Such an aversion would, I think, be an uncontroversial example of a bad motive. It might also interfere with what would otherwise be good motives. It might, for example, lead to a kind of subconscious bias in a person who is attempting to weigh up the claims of competing individuals as part of some reasoning process. Alternatively, it might limit the extent to which a person is able to feel sympathy for a member of the racial group in question… …Moreover, as with racial aversion,[aggression] could also interfere with good motives. It might, for example, cloud a person's mind in such a way that reasoning becomes difficult and the moral emotions are unlikely to be experienced.^29^

Douglas offers an account of moral enhancement which differs from that which we associate with Persson and Savulescu, as rather than increasing levels of moral emotion within an individual, Douglas’ approach would instead involve attenuation of *counter*‐moral emotions, such as those identified above. Douglas’ account of moral enhancement is therefore a clear example of where moral enhancement could align with commonly‐accepted elements of Kantian ethics through its attenuation of counter‐moral emotions to allow for greater capacity for moral reasoning. However, there could perhaps be some room for accounts of moral enhancement such as that of Persson and Savulescu (and perhaps even Kant's later approach of cultivation of certain emotions) as emotions such as empathy^30^ have also been shown to attenuate aggression.^31^

So then it seems as though there are grounds for us to say that moral bioenhancement through emotional modulation could in fact be permitted (or at the very least, not outright rejected) under a Kantian approach to ethics. However, there are arguments that could be raised here surrounding the fact that there could be a distinction made between more traditional approaches to moral enhancement (such as Kant's cultivation of sympathy through exposure to emotive stimuli) and biomedical means proposed under accounts of moral bioenhancement, which could even be considered artificial.^32^ For instance, there are concerns that cultivation of emotions such as sympathy beyond a certain point could lead to an overwhelming of the capacity for moral reasoning – as Denis writes: “All that Kantian morality can reasonably demand – and all that Kant thinks it demands – is that we not allow emotions to replace or hinder rational judgements.”^33^ De Lourdes Borges also notes that rationality remains at the centre of Kant's moral theory, even with this new role for sympathetic inclinations. As she notes:

The pathological emotions don't enable us to know when and where to apply moral principles, rather this is decided by the duty of humanity, which rationally figures out in which cases we should activate our natural sympathy and in which we should prevent it from arising. Pathological feelings, according to Kant, will be always blind to decide the right action in the right context…^34^

The concern that emotions “will always be blind to decide the right action in the right context” is echoed in moral enhancement literature. For instance, Robert Sparrow notes that, as morality and moral behaviour are context‐dependent, an automatic, visceral reaction to a moral dilemma or stimulus might not in fact lead to the correct moral action – even if the action is motivated by positive moral emotions. As Sparrow writes: “It would be a good drug, indeed, that made us feel love only for what is worthy of love and brave only in the service of a just cause.”^35^ Harris seconds this, asserting that no one can rely on their ‘moral nose’ and using it as further proof of the need for rationality in morality. As he puts it:

To believe that emotions can deliver answers to moral dilemmas or generate moral judgements is like believing that the gut is an organ of thought, or one that can answer complex, combined theoretical and empirical, questions… Ethical judgements cannot, literally cannot, be felt. There is no sense organ for such a feeling.^36^

This then further affirms the need for rationality and reason to remain at the centre of moral theory, and so even where a role for emotions such as sympathy in morality is acknowledged (as Kant himself even seemed to do), it can never be considered to play one that supersedes the role of reason.

So then, in cultivating our sympathetic inclinations we should take care to avoid cultivating them to the extent that they become overwhelming and so risk impairing agency and our ability to reason morally. But what would this mean for moral bioenhancement? Would augmentation of emotions such as sympathy by biomedical means (as opposed to the less dramatic means proposed above by Kant) lead to cultivation of these emotions at the cost of the ability to reason? Harris has voiced such concerns; arguing that adopting an emotional modulation approach to moral bioenhancement would put our capacity to reason about the moral situations in which we find ourselves at risk (and taking our freedom to do wrongly away along with it). As he puts it: “Emotional modulation is unlikely to leave us free to do what our intellect tells us is right if feelings of repugnance or emotional aversion are too strong to be routinely overridden.”^37^

However, it is not exactly clear that moral bioenhancement would have such a potent effect as to interrupt moral reason in the manner that Harris suggests. Indeed, where a person does demonstrate naturally (comparatively) high levels of empathy and sympathy, we do not consider them to be less able to engage in moral reasoning than their less‐sympathetic counterparts (a case made by many writers, such as DeGrazia,^38^ Rakić,^39^ and Persson and Savulescu^40^).

Further, speaking in reference to Kant's own writings, Baron asserts that, for Kant, we can only truly be overwhelmed by an emotion or desire if we have *chosen* to allow that emotion or desire to overwhelm us. She calls “the view that Kant believes that we are passive with respect to our feelings”^41^ an error, stating that:

…central to Kant's theory of agency is this thesis: “an incentive can determine the will to an action only so far as the individual has incorporated it into his maxim” (R 24/19). In other words, a desire does not simply overcome me; I act as the desire directs me, I do so because I decide to do so.^42^

And indeed, as noted above, it could be argued further that moral bioenhancement might actually aid our ability to reason morally by tempering those counter‐moral emotions that might otherwise hamper our capacity to do so.

It therefore seems that, perhaps contrary to expectation, moral enhancement would not be necessarily be *prima facie* dismissed or ruled out in Kantian ethics after all. However, Kant speaks of cultivating sympathy in a manner that we might associate with more traditional forms of moral enhancement^43^ (that is, as opposed to moral *bio*enhancement) in his suggestion that we seek out “places where the poor who lack the most basic necessities are to be found”.^44^ But it is less clear that this promotion would extend to moral *bio*enhancement; a much more controversial notion than traditional moral enhancement even in general discussion. In this context, it could be the case that bioenhancement of sympathy is considered artificial, and so it would be better for the agent to cultivate the emotion in the more traditional manner suggested by Kant. But this need not necessarily be the case, and indeed there may be some people on whom more traditional means of moral enhancement might have no effect^45^ meaning that moral bioenhancement would be their best (or even only) real hope of improving themselves morally. However the idea of introducing ‘artificial’ means to morally enhance agents neatly brings us to another relevant concern for Kantian ethicists: bodily integrity.

## 3 MIND, BODY, AND MORAL BIOENHANCEMENT

Kant strongly asserted that, as persons, we are necessarily embodied:

Our life is entirely conditioned by our body, so that we cannot conceive of a life not mediated by the body and we cannot make use of our freedom except through the body. [8]^46^

As embodied persons, we therefore have a particularly vested interest in what happens to that body; for instance, we cannot do anything degrading lest we lose our worth as persons. But it isn't necessarily clear *what* would qualify as a degrading act (and it is certainly unclear whether moral bioenhancement would count as such). As Chadwick puts it:

[Kant's] emphasis is thus on the preservation of human worth, and he depends on a notion of certain acts as intrinsically degrading. If we indulge in them we lose our worth. This view… is open to the objection that there is widespread disagreement as to what counts as degrading.^47^

However, Kant wrote that the mind must ensure that the body doesn't alter the state of the mind, that it must exercise control over the body to prevent this.

The body must first be disciplined, because in it there are *principia* by which the mind is affected, and through which the body alters the state of the mind. The mind must therefore take care to exercise an autocracy over the body, so that it cannot alter the state of the mind.^48^

This extract was most likely written with appetites and desires in mind, but there is perhaps some room for application for our purposes. Such an example would be the concern noted above, that undergoing moral bioenhancement could make us unable to think. Further, Harris argues that such an intervention could even remove our freedom to do wrong (which, echoing Milton's *Paradise Lost,* he refers to, as our ‘freedom to fall’).^49^ But as already noted, this would not necessarily be the case, moral bioenhancement interventions might not remove the freedom to fall, and indeed they might be something that simply quietens the mind of intrusive thoughts of an aggressive nature, better enabling us to reason. Or it could be that moral enhancement to strengthen or increase our levels of moral emotions such as sympathy would, as Marcia Baron suggests, in turn improve our ability to assess how best to help those in need.

Another concern that could be raised is that of identity; for if we are, as Kant suggests, necessarily embodied persons, then it could be argued that something which acts upon the body and that could thereafter have some effect on the mind (even if that effect would not necessarily impair reason), could be considered a threat to identity. Again, such a concern has been previously raised in the literature on both human and moral bioenhancement, as research by Riis, Simmons and Gordon demonstrated, “that healthy young people are more reluctant to enhance traits that are believed to be fundamental aspects of their self‐identities than traits that are believed to be less fundamental.”^50^ The most fundamental of the traits in question being those perhaps most likely to be affected by moral bioenhancement: empathy and kindness; as I have summarised elsewhere:

Riis and colleagues constructed a study where participants were given a list of 19 traits and were asked to rate how relevant each one was to self‐identity; participants were then asked to indicate whether they would be willing to enhance each trait. The results of two of the traits listed in the study are of particular significance for our purposes: empathy and kindness. These were regarded as being the most fundamental to self‐identity out of the 19 traits listed with only 13% and 9% (respectively) of the participants willing to enhance these traits—the lowest figures for the entire study.^51^

So then moral bioenhancement could perhaps be seen as a threat to identity, at least by some. However, it is not clear that this would necessarily be the case, especially if taking a more Kantian perspective – although only when the intervention is freely chosen by the agent, and so not forced upon him by law or medicine. As Korsgaard explains:

If I can overcome my cowardice by surgery or medication rather than habituation I might prefer to take this less arduous route. So long as an authentic good will is behind my desire for greater courage, and authentic courage is the result, the mechanism should not matter. But for the Kantian it does matter who is initiating the use of the mechanism. Where I change myself, the sort of continuity needed for identity may be preserved, even if I become very different. Where I am changed by wholly external forces, it is not.^52^

In any case, despite the concerns already noted, there could still be room to allow for moral bioenhancement regardless under certain circumstances, as the motive of self‐preservation could, for Kant, provide justification for certain bodily interventions which might otherwise have been considered impermissible.^53^ So I could, for example, amputate a foot that was becoming gangrenous in order to save my life. This exception could also allow moral bioenhancement: Imagine a man whose excessive aggression frequently causes him to find himself in dangerous situations; getting into bar fights and the like. He knows that if he weren't so aggressive, he wouldn't find himself in these situations^54^ and that there is always a risk that he won't come out of the next fight alive. But his aggression overtakes him, he cannot control it, and he finds himself in these situations again and again. Such an example also echoes the Kantian concept of a mental disorder of the affects, where intensity of feeling overpowers the ability to reflect. As Frierson writes:

One might murder someone out of rage, but one's power of choice is not involved… Acting or failing to act due to affect is morally similar to acting or failing to act when one is asleep. In both cases one is morally unconscious, so to speak, rather than morally corrupt.^55^

Surely then, this would be an example where moral bioenhancement could be used in the name of self‐preservation. In attenuating his aggression^56^ the man in question finds that he no longer throws himself into these dangerous situations, no longer provokes fights, and so on. And, as a result, the risk to his life is greatly reduced. Moral bioenhancement has been used to secure his self‐preservation.

However, this seems to suggest that moral bioenhancement could only be justified under very specific circumstances, perhaps only for those people for whom it might be considered medically indicated.^57^ This could indeed be the case, if we accept the concerns relating to bodily integrity as noted above then moral bioenhancement seems to be only acceptable in instances of self‐preservation. However it is not clear that these concerns are founded: I have already noted that augmented moral emotions (or tempered counter‐moral emotions) would be unlikely to impair, and could in fact aid, our moral reasoning. Further, as regards the question of the impact of identity, it might be the case that the respondents in the research by Riis and colleagues were simply mistaken, and that moral bioenhancement would have no real or fundamental impact on their identities, especially if (as Korsgaard argued) the enhancement intervention was freely‐chosen.

## 4 MORAL BIOENHANCEMENT AND DUTY

It seems from our discussion so far that moral enhancement interventions could aid us in behaving morally and possibly in becoming more moral persons. From this it seems that perhaps moral enhancement could therefore help us to fulfil certain Kantian duties where particular importance is placed on behaving morally and on being a moral person, such as the duty of moral perfection. But could this be the case? And if indeed moral enhancement would help us to fulfil such a duty, then could this in turn mean that there is a duty to undergo the intervention?

The duty to strive for moral perfection is a duty to self, a concept that is not entirely uncontroversial. I will explain and defend this concept briefly before discussing the duty to strive for moral perfection (and its relation to moral enhancement interventions) in particular. As Denis explains, duties to self:

…comprise a varied collection of commands and prohibitions. They prohibit willing (determining oneself to action) on maxims (subjective to practical principles) corresponding to the vices of suicide, sexual self‐degradation, gluttony and drunkenness, lying, avarice, and servility. These duties also require having the goals of moral and natural perfection.^58^

The idea of having a set of duties to oneself is not without its critics, many of whom cite the (supposed) other‐facing nature of morality. As Denis notes: “Proponents of this view often presuppose that we naturally look out for our own well‐being, so morality's purpose is to get us to care about the well‐being of others.”^59^

Kantian ethics is of course other‐regarding, due in no small part to the second categorical imperative: the Formula of Humanity. This formulation can be described in simple terms as the duty to ensure that we never treat others as a means to an end, only ever as an end in themselves. While Kantian ethics is other‐regarding, however, it is not *solely* other‐regarding, and it is this same formulation that also lays the path for self‐regarding duties. For Kant expresses the formula of humanity in the following manner: “Act so that you treat humanity, whether *in your own person* or in the person of another, always at the same time as an end, and never only as a means.”^60^ So here we can clearly see that the formula is also self‐regarding (as indicated by my emphasis above) as well as other‐regarding. Further, just as others are rational creatures deserving of dignity, so too are we. Denis notes that “no being with dignity has greater value than any other being with dignity.”^61^ So then, as I have dignity, I have equal value with other people who have dignity as well. This lays the grounding for duties to self in general, and so the duty to strive for moral perfection as well.

Having established the legitimacy of duties to self, such as the duty to strive for moral perfection, both for Kant and in more general terms, it is important to clarify what the duty to strive for moral perfection itself actually involves (and from there assess whether moral enhancement interventions could be said to help us to fulfil it). Allen Wood asserts that moral perfection “includes not only the inner strength that makes us immune to affects but also the cultivation of inclinations which add to the strength of our good maxims.”^62^ So then, according to Wood, moral perfection involves being able to avoid being steered mindlessly by emotions and inclinations, but also involves cultivating those inclinations that actually aid us in behaving morally. This view seems to be seconded by Denis, who writes:

The virtuous Kantian agent blocks affects from hindering her deliberation. She refuses to let her passions or inclinations determine her will. Yet she does not think that emotions as such are contrary to morality: she distinguishes healthy susceptibility to feelings – which can aid her in recognizing morally salient aspects of situations – from the tendency to indulge indiscriminately in her feelings to no practical purpose.^63^

As already discussed above, moral enhancement would most likely increase levels of those moral emotions (such as sympathy) which Kant asserts that we should cultivate, and which Wood notes as a component of moral perfection. Further, the intervention could even help us to temper less helpful affects, such as aggression, and so prevent them from impairing our ability to reason morally. Therefore, it seems that moral enhancement interventions could indeed help us to strive for moral perfection – and so would aid us to fulfil the duty to do so.

Further, the duty to strive for moral perfection is an imperfect duty; in short, this means that while the end (of moral perfection) remains fixed, the actions to promote that end are not specified. As Moran explains:

According to Kant, duties of wide obligation – like imperfect duties – are “duties that prescribe the maxims of actions, not the actions themselves” or that leave “playroom for free choice in following the law”.^64^

These are in contrast to perfect duties, which prohibit maxims of action such as (for example) lying and suicide and unlike imperfect duties, they permit “no exception in the interest of inclination.”^65^ So then, as the duty to strive for moral perfection is an imperfect duty, we have free choice in what action we take to pursue it; moral bioenhancement could be one such choice.

It therefore seems that moral enhancement may indeed help us to fulfil the imperfect duty to strive for moral perfection. But is this enough to qualify moral enhancement as a duty in its own right? Surely to be considered as such, moral enhancement would, as noted below, have to be the best way to fulfil this duty to strive for moral perfection – and it is not necessarily clear that it is. Rather, it is much more likely to be the case that moral enhancement is “simply one way among many to fulfil our duty of moral perfection”.^66^^,^ 67 This position is backed up separately by Brassington, who – in referencing the writings of Harris regarding a duty to enhance^68^ (that is, human enhancement more generally rather than moral enhancement) – writes: “…even if we do have a duty to make the world a better place, it does not follow that everything that makes the world a better place is a duty.”^69^ Similarly then, even if we do have a duty to strive for moral perfection, it does not necessarily follow that everything that helps us towards this goal is itself a duty.

However, might there be an exception here in the case of people who may otherwise be incapable of fulfilling the duty of moral perfection? In this instance, could moral enhancement perhaps be considered the best possible way to fulfil the duty in question, and so a duty in its own right? Perhaps there is room to suggest that there *could* be a duty to undergo moral enhancement, even if only in the case of those who otherwise could not fulfil their duties or pursue the end of moral perfection (however small a group that might be). In such instances, the maxim could be universalised that: *any persons whose inability to fulfil their duty of moral perfection is rooted in a condition* 70 *that could be remedied by moral enhancement interventions, should undergo moral enhancement*.

## 5 CONCLUSION

Perhaps contrary to what might otherwise be assumed at first glance, and maybe even at odds with the polarization of the rational and emotional approaches to morality (which Kant seemed to embody in his conflict with Hume), moral bioenhancement by emotional modulation could in fact be permissible under a Kantian approach to ethics. With the role of emotions such as sympathy in morality acknowledged by Kant himself, and the recognition that there are people whose emotional make‐up is such that they are otherwise unable to behave morally unless provided with an opportunity to enhance their levels of empathy, a place for moral bioenhancement in Kantian ethics becomes more and more apparent. Further, although this endeavour would be unlikely to be considered as such for the vast majority of moral agents, for a small number of people, undergoing moral bioenhancement could even amount to a duty.

## ORCID

*Sarah Carter* http://orcid.org/0000-0002-3619-8640

## Footnotes

1
According to the philosopher Allen Wood: see Wood, A. (2009). Duties to oneself, duties of respect to others. In T. E. Hill Jr. (Ed.), *The Blackwell Guide to Kant's Ethics* (pp. 229–251). Oxford: Wiley‐Blackwell Publishing.

2
A topic which is explored in more depth in this article.

3
Hume, D. (2005). Treatise of human nature. In M. L. Morgan (Ed.), *Classics of Moral and Political Philosophy* (pp. 752–774). USA: Hackett Publishing Company Ltd.

4
Denis, L., & Wilson, E. (2016). *The Stanford Encyclopedia of Philosophy: Kant and Hume on Morality* Retrieved from http://plato.stanford.edu/archives/fall2016/entries/kant-hume-morality/

5
I will use the terms “enhancement” and “bioenhancement” as interchangeable unless otherwise stated.

6
Douglas, T. (2008). Moral enhancement. *Journal of Applied Philosophy*, *25*(3), 229–345. 10.1111/j.1468-5930.2008.00412.xPMC261468019132138

7
Persson, I., & Savulescu, J. (2012). *Unfit for the future: The need for moral enhancement*. Oxford, Oxford University Press.

8
Harris, J. (2013). “Ethics is for bad guys!” Putting the ‘moral’ into moral enhancement. *Bioethics*, *27*(3), 169–173. 10.1111/j.1467-8519.2011.01946.x22296666

9
It is this emotion‐centred account of moral bioenhancement which will be the focal point of this article, in particular the account offered by Persson and Savulescu (that moral bioenhancement would involve amplification of empathy). As such, any mention of moral bioenhancement from this point onwards, unless otherwise stated, will be in reference to this account.

10
Which, as I will note, may in fact be a reference to what we today call empathy.

11
At least in certain circumstances.

12
As noted above; see also Wood, *op. cit.* note 1, p. 243.

13
Moran, K. A. (2012). *Community and progress in Kant's moral philosophy* (p. 200). USA: Catholic University of America Press.

14
Again, a more detailed explanation of this distinction is found later in this article.

15
As noted below. See also: Baron, M. W. (1995). *Kantian ethics almost without apology*. New York: Cornell University Press.

16
Wispé, L. (1987). History of the concept of empathy. In N. Eisenberg & J. Strayer (Eds.), *Empathy and its Development* (pp. 17–37). USA: Cambridge University Press.

17
Hodges, S. D. & Klein, K. J. K. (2001). Regulating the costs of empathy: The price of being human. *Journal of Socio‐Economics*, *30*(5), 437–452.

18
Wispé, *op. cit.* note 16.

19
For instance, Persson & Savulescu, *op. cit.* note 7.

20
As cited in Baron, *op. cit.* note 15, p. 199.

21
De Lourdes Borges, M. (2002). Kant on sympathy and moral motives. *Ethic@: An International Journal for Moral Philosophy*, *1*(2), 183–199.

22
Baron, *op. cit.* note 15.

23
De Lourdes Borges, *op. cit.* note 21, p. 194.

24
Kant, I. (2001). Of duties in regard to the body itself. In P. Heath & J. B. Schneewind (Eds.), *Lectures on Ethics* (pp. 151–153). USA: Cambridge University Press.

25
De Lourdes Borges, *op. cit.* note 21, p. 188.

26
Baron, *op. cit.* note 15.

27
Denis, L. (1997). Kant's ethics and duties to oneself. *Pacific Philosophical Quarterly*, *78*(4), 321–348.

28
Douglas, *op. cit.* note 6, pp. 229–345.

29
Ibid: 231.

30
Which, as I have noted above, is linked to sympathy.

31
Persson, I., & Savulescu, J. (2013). Getting moral enhancement right: the desirability of moral bioenhancement. *Bioethics*, *27*(3), 124–131. 10.1111/j.1467-8519.2011.01907.xPMC337847021797913

32
A concern on which I shall expand in the following section.

33
Douglas, *op. cit.* note 6, p. 341.

34
De Lourdes Borges, *op. cit.* note 21, p. 195.

35
Sparrow, R. (2014). Better living through chemistry? A reply to Savulescu and Persson on 'Moral Enhancement'. *Journal of Applied Philosophy*, *31*(1), 23–32.

36
Harris, J. (2013). Moral progress and moral enhancement. *Bioethics*, *27*(5), 285–290. 10.1111/j.1467-8519.2012.01965.x22712524

37
Harris, J. (2012). What It's like to be good. *Cambridge Quarterly of Healthcare Ethics*, *21*(3), 293–305. 10.1017/S096318011100086722261368

38
DeGrazia, D. (2014). Moral enhancement, freedom, and what we (should) value in moral behaviour. *Journal of Medical Ethics*, *40*(6), 361–368. 10.1136/medethics-2012-10115723355049

39
Rakić, V. (2014). Voluntary moral enhancement and the survival‐at‐any‐cost bias. *Journal of Medical Ethics*, *40*(4), 246–250. 10.1136/medethics-2012-10070023412695

40
Persson, I. & Savulescu, J. (2012). Moral enhancement, freedom and the God machine. *Monist, 95*(3), 399–421. 10.5840/monist201295321PMC343113022942461

41
Baron, *op. cit.* note 15, p. 196.

42
Ibid.

43
Which typically involves things such as moral education and raising awareness of moral issues.

44
As quoted in Baron, *op. cit.* note 15, p. 196.

45
I offer a more detailed account of this concern in Carter, S. (2016). Could moral enhancement interventions be medically indicated? *Health Care Analysis*, doi: 10.1007/s10728-016-0320-8. 10.1007/s10728-016-0320-8PMC565672926910729

46
As cited in Chadwick, R. F. (1989). The market for bodily parts: Kant and duties to oneself. *Journal of Applied Philosophy*, *6*(2), 129–140.

47
Ibid: p. 130.

48
Kant, *op. cit.* note 24, p. 151.

49
Harris, *op. cit.* note 36. There is some dispute as to whether this is a freedom worth protecting, but Harris insists that it is, writing: “Without the freedom to fall, good cannot be a choice; and freedom disappears and along with it virtue. There is no virtue in doing what you must.” Harris, J. (2011). Moral enhancement and freedom. *Bioethics*, *25*(2), 104.

50
Riis, J., Simmons, J. P., Goodwin, G. P. (2008). Preference for enhancement pharmaceuticals: the reluctance to enhance fundamental traits. *Journal of Consumer Research*, *35*, 495–508.

51
Carter, S. (2015). Putting a price on empathy: against incentivising moral enhancement. *Journal of Medical Ethics*, *41*(10), 825–829. 10.1136/medethics-2015-10280426265725

52
Korsgaard, C. (1989). Personal Identity and the unity of agency: a Kantian response to Parfit. *Philosophy & Public Affairs*, *18*(2), 101–132.

53
Chadwick, *op. cit.* note 46, pp. 129–140.

54
This is not to say that *only* excessively aggressive people find themselves in these situations, but it does seem reasonable to assume that persons with an excess of aggression would be more likely to find themselves in such situations with far more regularity than those without.

55
Frierson, P. (2009). Kant on mental disorder. Part 2: Philosophical implications of Kant's account. *History of Psychiatry*, *20*(3), 290–310. 10.1177/0957154X0833764420030161

56
As noted above, empathy can also temper aggression, so emotional augmentation by moral bioenhancement would also qualify here in addition to the account of the endeavour offered by Douglas (see Douglas, *op. cit*. note 6).

57
See Carter, *op. cit.* note 45.

58
Denis, L. (1997). *op. cit.* note 27, p. 324.

59
Ibid: 322.

60
Ibid: 324 (my emphasis).

61
Ibid: 325.

62
Wood, *op. cit.* note 1, p. 243.

63
Denis, *op. cit.* note 58.

64
Moran, *op. cit.* note 13.

65
Denis, *op. cit.* note 58, p. 326.

66
Moran, *op. cit.* note 13.

67
This comment was made in reference to the role of friendship in Kantian ethics, however the argument still stands.

68
Harris, J. (2007). *Enhancing evolution*. Oxfordshire: Princeton University Press.

69
Brassington, I. (2010). Enhancing evolution and enhancing evolution. *Bioethics*, *24*(8), 395–402. 10.1111/j.1467-8519.2008.01703.x19222450

70
Such as a deficit of empathy.

